# (*E*)-1-(2-Iodo­phen­yl)-2-phenyl­diazene

**DOI:** 10.1107/S1600536811032119

**Published:** 2011-08-11

**Authors:** David S. Wragg, Mohammed A. K. Ahmed, Ola Nilsen, Helmer Fjellvåg

**Affiliations:** a inGAP Centre for Research Based Innovation, Center for Materials Science and Nanotechnology, Department of Chemistry, University of Oslo, PO Box 1033 Blindern, Oslo 0315, Norway; b Department of Chemistry, University of Oslo, PO Box 1033 Blindern, Oslo 0315, Norway

## Abstract

The mol­ecule of the title compound, C_12_H_9_IN_2_, is approximately planar [maximum deviation = 0.020 (5) Å] with a *trans* arrangement of the groups around the N=N double bond. This double bond is rotated away from the iodine substiuent.

## Related literature

For the synthesis, see: Badger *et al.* (1964 ▶).
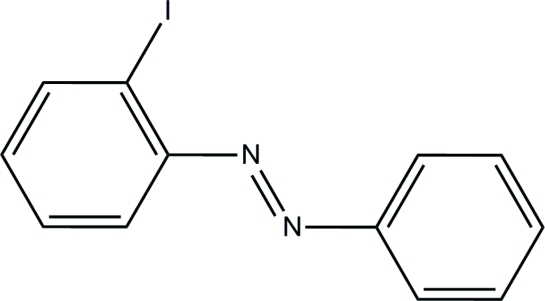


## Experimental

### 

#### Crystal data

C_12_H_9_IN_2_
*M* *_r_* = 308.11
Orthorhombic, 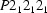

*a* = 4.628 (3) Å
*b* = 12.801 (9) Å
*c* = 18.312 (12) Å
*V* = 1084.9 (13) Å^3^
*Z* = 4
Mo *K*α radiation
μ = 2.92 mm^−1^
*T* = 296 K
1.00 × 0.07 × 0.07 mm

#### Data collection

Bruker APEXII CCD diffractometer
Absorption correction: multi-scan (*SADABS*; Bruker, 2011 ▶) *T*_min_ = 0.783, *T*_max_ = 0.822
10050 measured reflections
1930 independent reflections
1842 reflections with *I* > 2σ(*I*)
*R*_int_ = 0.033

#### Refinement

*R*[*F* ^2^ > 2σ(*F* ^2^)] = 0.028
*wR*(*F* ^2^) = 0.070
*S* = 1.07
1930 reflections
137 parameters
H-atom parameters constrained
Δρ_max_ = 0.57 e Å^−3^
Δρ_min_ = −0.48 e Å^−3^
Absolute structure: Flack (1983 ▶), 763 Friedel pairs
Flack parameter: 0.08 (4)

### 

Data collection: *APEX2* (Bruker, 2011 ▶); cell refinement: *SAINT* (Bruker, 2011 ▶); data reduction: *SAINT*; program(s) used to solve structure: *SHELXS97* (Sheldrick, 2008 ▶); program(s) used to refine structure: *SHELXL97* (Sheldrick, 2008 ▶) in *WinGX* (Farrugia, 1999 ▶); molecular graphics: *DIAMOND* (Brandenburg, 2006 ▶); software used to prepare material for publication: *PLATON* (Spek, 2009 ▶) and *publCIF* (Westrip, 2010 ▶).

## Supplementary Material

Crystal structure: contains datablock(s) I, global. DOI: 10.1107/S1600536811032119/qm2021sup1.cif

Structure factors: contains datablock(s) I. DOI: 10.1107/S1600536811032119/qm2021Isup2.hkl

Supplementary material file. DOI: 10.1107/S1600536811032119/qm2021Isup3.cml

Additional supplementary materials:  crystallographic information; 3D view; checkCIF report

## Figures and Tables

**Fig. 1.**
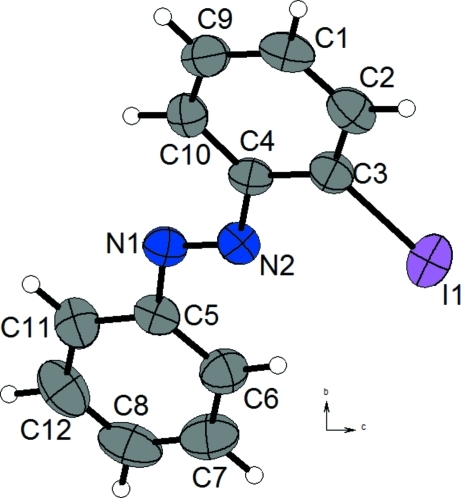
The asymmetric unit of (1) shown with thermal ellipsoids at 50% probability. Hydrogen atoms unlabelled for clarity.

**Fig. 2.**
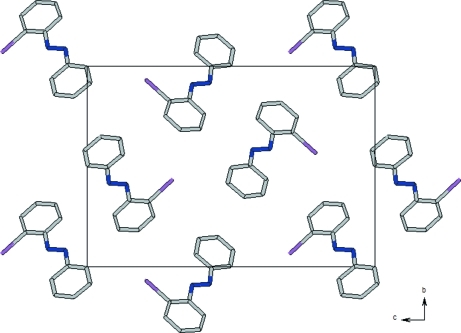
Packing diagram of (1) viewed along the a-axis.

**Fig. 3.**
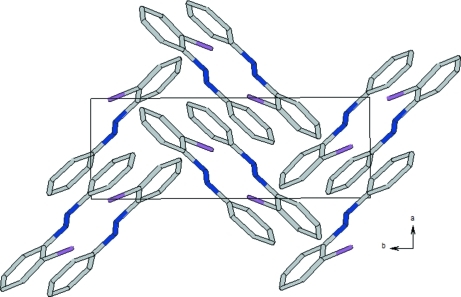
Packing diagram of (1) viewed along the c-axis.

## supplementary crystallographic information

### Comment

(*E*)-1-(2-iodophenyl)-2-phenyldiazene (1) was synthesized by a literature procedure (Badger *et al.* 1964) and recrystallized from absolute ethanol. The molecule is planar with a *trans* arrangement of the phenyl groups around the N—N double bond. This double bond is rotated away from the iodine substiuent on C3 (Fig. 1). There are no strong intermolecular interactions, although π-stacking interactions may exist between the phenyl rings and the N—N double bonds (Fig. 2, 3).

### Experimental

(1) was synthesized according to the literature procedure (Badger *et al.* 1964) and recrystallized from absolute ethanol in the form of long orange needles.

### Refinement

Least squares refinement was carried out with *SHELXL97* (Sheldrick, 2008) as implemented in *WinGX* (Farrugia, 1999). Hydrogen atoms were refined using a riding model the *U*_iso_ set to 1.2 times that of the heavy atom to which they are attached.

### Crystal data

**Table:**

C_12_H_9_IN_2_	*F*(000) = 592
*M**_r_* = 308.11	*D*_x_ = 1.886 Mg m^−^^3^
Orthorhombic, *P*2_1_2_1_2_1_	Mo *K*α radiation, λ = 0.71073 Å
Hall symbol: P 2ac 2ab	Cell parameters from 4258 reflections
*a* = 4.628 (3) Å	θ = 2.2–25.0°
*b* = 12.801 (9) Å	µ = 2.92 mm^−^^1^
*c* = 18.312 (12) Å	*T* = 296 K
*V* = 1084.9 (13) Å^3^	Needle, orange
*Z* = 4	1.00 × 0.07 × 0.07 mm

### Data collection

**Table:**

Bruker APEXII CCD diffractometer	1930 independent reflections
Radiation source: sealed tube	1842 reflections with *I* > 2σ(*I*)
graphite	*R*_int_ = 0.033
φ and ω scans	θ_max_ = 25.1°, θ_min_ = 1.9°
Absorption correction: multi-scan (*SADABS*; Bruker, 2011)	*h* = −5→5
*T*_min_ = 0.783, *T*_max_ = 0.822	*k* = −15→15
10050 measured reflections	*l* = −21→21

### Refinement

**Table:**

Refinement on *F*^2^	Secondary atom site location: difference Fourier map
Least-squares matrix: full	Hydrogen site location: inferred from neighbouring sites
*R*[*F*^2^ > 2σ(*F*^2^)] = 0.028	H-atom parameters constrained
*wR*(*F*^2^) = 0.070	*w* = 1/[σ^2^(*F*_o_^2^) + (0.0345*P*)^2^ + 0.6387*P*] where *P* = (*F*_o_^2^ + 2*F*_c_^2^)/3
*S* = 1.07	(Δ/σ)_max_ = 0.001
1930 reflections	Δρ_max_ = 0.57 e Å^−^^3^
137 parameters	Δρ_min_ = −0.48 e Å^−^^3^
0 restraints	Absolute structure: Flack (1983), 763 Friedel pairs
Primary atom site location: structure-invariant direct methods	Flack parameter: 0.08 (4)

### Special details

**Table:**

Geometry. All s.u.'s (except the s.u. in the dihedral angle between two l.s. planes) are estimated using the full covariance matrix. The cell s.u.'s are taken into account individually in the estimation of s.u.'s in distances, angles and torsion angles; correlations between s.u.'s in cell parameters are only used when they are defined by crystal symmetry. An approximate (isotropic) treatment of cell s.u.'s is used for estimating s.u.'s involving l.s. planes.
Refinement. Refinement of *F*^2^ against ALL reflections. The weighted *R*-factor *wR* and goodness of fit *S* are based on *F*^2^, conventional *R*-factors *R* are based on *F*, with *F* set to zero for negative *F*^2^. The threshold expression of *F*^2^ > 2σ(*F*^2^) is used only for calculating *R*-factors(gt) *etc*. and is not relevant to the choice of reflections for refinement. *R*-factors based on *F*^2^ are statistically about twice as large as those based on *F*, and *R*- factors based on ALL data will be even larger.

### Fractional atomic coordinates and isotropic or equivalent isotropic displacement parameters (Å^2^)

**Table:**

	*x*	*y*	*z*	*U*_iso_*/*U*_eq_	
C1	0.1092 (10)	0.3166 (4)	0.1768 (3)	0.0549 (12)	
H1	−0.0297	0.3672	0.1865	0.066*	
C2	0.1881 (10)	0.2473 (3)	0.2300 (3)	0.0521 (11)	
H2	0.1032	0.2496	0.2760	0.062*	
C3	0.3968 (9)	0.1737 (3)	0.2138 (2)	0.0450 (10)	
C4	0.5235 (10)	0.1691 (3)	0.1465 (2)	0.0389 (8)	
C5	1.0496 (8)	0.0040 (3)	0.0680 (2)	0.0407 (9)	
C6	1.1155 (10)	−0.0629 (3)	0.1240 (3)	0.0530 (10)	
H6	1.0261	−0.0561	0.1693	0.064*	
C7	1.3160 (11)	−0.1401 (4)	0.1121 (3)	0.0643 (13)	
H7	1.3637	−0.1860	0.1495	0.077*	
C8	1.4446 (10)	−0.1497 (4)	0.0459 (3)	0.0637 (14)	
H8	1.5794	−0.2025	0.0383	0.076*	
C9	0.2346 (10)	0.3116 (3)	0.1090 (3)	0.0523 (11)	
H9	0.1763	0.3584	0.0731	0.063*	
C10	0.4372 (8)	0.2417 (3)	0.0934 (2)	0.0420 (10)	
H10	0.5218	0.2408	0.0473	0.050*	
C11	1.1796 (10)	−0.0058 (4)	0.0017 (3)	0.0550 (12)	
H11	1.1331	0.0400	−0.0360	0.066*	
C12	1.3793 (10)	−0.0833 (4)	−0.0095 (3)	0.0638 (14)	
H12	1.4693	−0.0903	−0.0546	0.077*	
N1	0.8420 (7)	0.0855 (3)	0.07490 (19)	0.0433 (8)	
N2	0.7314 (7)	0.0892 (3)	0.13658 (19)	0.0422 (8)	
I1	0.51175 (8)	0.06841 (2)	0.294862 (15)	0.06222 (13)	

### Atomic displacement parameters (Å^2^)

**Table:**

	*U*^11^	*U*^22^	*U*^33^	*U*^12^	*U*^13^	*U*^23^
C1	0.050 (2)	0.039 (2)	0.075 (3)	0.0054 (19)	0.000 (2)	−0.010 (2)
C2	0.048 (3)	0.052 (3)	0.056 (3)	−0.006 (2)	0.009 (2)	−0.013 (2)
C3	0.045 (2)	0.042 (2)	0.048 (2)	−0.0057 (17)	−0.0028 (18)	−0.0042 (18)
C4	0.036 (2)	0.0332 (16)	0.0476 (19)	0.003 (2)	0.000 (2)	−0.0029 (13)
C5	0.032 (2)	0.039 (2)	0.051 (2)	0.0013 (18)	−0.0029 (18)	−0.0058 (15)
C6	0.051 (2)	0.050 (2)	0.058 (3)	0.005 (2)	0.001 (2)	0.002 (2)
C7	0.056 (3)	0.051 (3)	0.086 (4)	0.011 (2)	−0.006 (3)	0.006 (3)
C8	0.043 (3)	0.048 (2)	0.100 (4)	0.008 (2)	−0.007 (3)	−0.021 (2)
C9	0.055 (3)	0.042 (2)	0.060 (3)	−0.002 (2)	−0.013 (2)	0.003 (2)
C10	0.038 (2)	0.047 (2)	0.041 (2)	−0.0067 (18)	−0.0007 (17)	−0.0027 (16)
C11	0.054 (3)	0.063 (3)	0.049 (3)	0.004 (2)	−0.001 (2)	−0.005 (2)
C12	0.046 (2)	0.079 (4)	0.066 (3)	0.005 (3)	0.003 (2)	−0.025 (3)
N1	0.0426 (18)	0.040 (2)	0.047 (2)	0.0022 (16)	0.0011 (16)	−0.0016 (15)
N2	0.0394 (17)	0.044 (2)	0.0433 (19)	−0.0003 (15)	0.0006 (15)	−0.0031 (15)
I1	0.0674 (2)	0.0688 (2)	0.05044 (18)	−0.0007 (2)	0.0006 (2)	0.01231 (12)

### Geometric parameters (Å, °)

**Table:**

C1—C2	1.367 (7)	C6—H6	0.9300
C1—C9	1.371 (7)	C7—C8	1.356 (7)
C1—H1	0.9300	C7—H7	0.9300
C2—C3	1.381 (6)	C8—C12	1.357 (7)
C2—H2	0.9300	C8—H8	0.9300
C3—C4	1.367 (6)	C9—C10	1.328 (6)
C3—I1	2.075 (4)	C9—H9	0.9300
C4—C10	1.403 (5)	C10—H10	0.9300
C4—N2	1.416 (5)	C11—C12	1.371 (7)
C5—C11	1.361 (6)	C11—H11	0.9300
C5—C6	1.370 (6)	C12—H12	0.9300
C5—N1	1.424 (5)	N1—N2	1.241 (5)
C6—C7	1.374 (6)		
			
C2—C1—C9	120.1 (4)	C8—C7—H7	119.9
C2—C1—H1	120.0	C6—C7—H7	119.9
C9—C1—H1	120.0	C7—C8—C12	120.9 (4)
C1—C2—C3	118.5 (4)	C7—C8—H8	119.5
C1—C2—H2	120.8	C12—C8—H8	119.5
C3—C2—H2	120.8	C10—C9—C1	121.7 (4)
C4—C3—C2	121.5 (4)	C10—C9—H9	119.1
C4—C3—I1	120.5 (3)	C1—C9—H9	119.1
C2—C3—I1	118.0 (3)	C9—C10—C4	119.9 (4)
C3—C4—C10	118.3 (4)	C9—C10—H10	120.1
C3—C4—N2	116.0 (3)	C4—C10—H10	120.1
C10—C4—N2	125.7 (4)	C5—C11—C12	119.8 (5)
C11—C5—C6	120.8 (4)	C5—C11—H11	120.1
C11—C5—N1	116.4 (4)	C12—C11—H11	120.1
C6—C5—N1	122.8 (4)	C8—C12—C11	119.5 (5)
C5—C6—C7	118.7 (5)	C8—C12—H12	120.3
C5—C6—H6	120.6	C11—C12—H12	120.3
C7—C6—H6	120.6	N2—N1—C5	112.8 (3)
C8—C7—C6	120.3 (5)	N1—N2—C4	115.1 (3)
			
C9—C1—C2—C3	0.6 (7)	C1—C9—C10—C4	1.3 (7)
C1—C2—C3—C4	−0.1 (6)	C3—C4—C10—C9	−0.8 (6)
C1—C2—C3—I1	−179.5 (3)	N2—C4—C10—C9	178.5 (4)
C2—C3—C4—C10	0.3 (6)	C6—C5—C11—C12	−0.1 (7)
I1—C3—C4—C10	179.6 (3)	N1—C5—C11—C12	−179.2 (4)
C2—C3—C4—N2	−179.1 (4)	C7—C8—C12—C11	−0.3 (8)
I1—C3—C4—N2	0.2 (5)	C5—C11—C12—C8	0.1 (7)
C11—C5—C6—C7	0.1 (7)	C11—C5—N1—N2	179.6 (4)
N1—C5—C6—C7	179.1 (4)	C6—C5—N1—N2	0.5 (5)
C5—C6—C7—C8	−0.2 (7)	C5—N1—N2—C4	179.9 (3)
C6—C7—C8—C12	0.3 (8)	C3—C4—N2—N1	179.3 (4)
C2—C1—C9—C10	−1.2 (7)	C10—C4—N2—N1	0.0 (6)

## References

[bb1] Badger, G. M., Drewer, R. J. & Lewis, G. E. (1964). *Aust. J. Chem.* **17**, 1036–1049.

[bb2] Brandenburg, K. (2006). *DIAMOND* Crystal Impact GbR, Bonn, Germany.

[bb3] Bruker (2011). *APEX2*, *SAINT* and *SADABS* Bruker AXS Inc., Madison, Wisconsin, USA.

[bb4] Farrugia, L. J. (1999). *J. Appl. Cryst.* **32**, 837–838.

[bb5] Flack, H. D. (1983). *Acta Cryst.* A**39**, 876–881.

[bb6] Sheldrick, G. M. (2008). *Acta Cryst.* A**64**, 112–122. 10.1107/S0108767307043930 18156677

[bb7] Spek, A. L. (2009). *Acta Cryst.* D**65**, 148–155. 10.1107/S090744490804362X PMC2631630 19171970

[bb8] Westrip, S. P. (2010). *J. Appl. Cryst.* **43**, 920–925.

